# Supplementary material for: The HCN domain couples voltage gating and cAMP response in hyperpolarization-activated cyclic nucleotide-gated channels
Source: eLife. 2019 Nov 26;8:e49672. doi: 10.7554/eLife.49672 (PMC6894927; doi:10.7554/eLife.49672)
Supplement: Supplementary file 1. — (A) Fitting parameters of the activation curves in HCN2 (Figures 2,4,6,7). From left to right: half-activation voltage (V1/2), inverse slope factor (k) obtained by fitting data to a Boltzmann function (Material and methods) in absence or presence of cAMP; n = number of cell tested in each condition; cAMP-induced shift in V1/2; number of cells that expressed a measurable HCN current. *p<0.05 by One-way ANOVA with Fisher’s test compared to wt HCN2; §p<0.05 by Student’s T-test compared to control condition (without cAMP); n.s. not statistically different; n.t. not tested; n.d. not detectable. cAMP concentration was 15 µM in all cases, except for last row (HCN2 K464-E478A#, 100 µM cAMP). (B) Fitting parameters of the activation curves in HCN1 and HCN4 (Figures 2,7). From left to right: half-activation voltage (V1/2), inverse slope factor (k) obtained by fitting data to a Boltzmann function (Material and methods) in absence or presence of cAMP; n = number of cell tested in each condition; cAMP-induced shift in V1/2; number of cells that expressed a measurable HCN current. *p<0.05 by One-way ANOVA with Fisher’s test compared to wild-type HCN1 or HCN4; §p<0.05 by Student’s T-test compared to control condition (without cAMP); n.s. not statistically different; n.t. not tested; n.d. not detectable. cAMP concentration used for HCN1 and HCN4 was 15 µM and 30 µM respectively. (C) Number of molecules for each simulation of molecular dynamics performed on HCN1. POPC: 1-palmitoyl-2-oleoyl-glycero-3-phosphocholine; TIP3P: water model; K: K+ ion; CL: Cl- ion [file elife-49672-supp1.docx]

**Supplementary file 1A**

Fitting parameters of the activation curves in HCN2.

From left to right: half-activation voltage (V_1/2_) , inverse slope factor (k) obtained by fitting data to a Boltzmann function (Material and Methods) in absence or presence of cAMP; n= number of cell tested in each condition; cAMP-induced shift in V_1/2_; number of cells that expressed a measurable HCN current.

*p<0.05 by One-way ANOVA with Fisher’s test compared to wt HCN2; ^§^p<0.05 by Student’s T-test compared to control condition (without cAMP); n.s. not statistically different; n.t. not tested; n.d. not detectable. cAMP concentration was 15 µM in all cases, except for last row (HCN2 K464-E478A^#^, 100 µM cAMP).

|  | **V_1/2_ (mV)**  **± SEM** | **k (mV)**  **± SEM** | **n** | **V_1/2_ (mV)**  **± SEM with cAMP** | **k (mV)**  **± SEM with cAMP** | **n** | **V_1/2_ shift due to cAMP (mV)**  **± SEM** | **Number of cells expressing current** |
| --- | --- | --- | --- | --- | --- | --- | --- | --- |
| **HCN2 wt** | - 95.3 ± 0.5 | 6 ± 0.5 | 28 | n.t. | n.t. | - | - | 28/28 |
| **HCN2 I177V** | - 93.8 ± 0.6 ^n.s.^ | 5.5 ± 0.5 | 6 | n.t. | n.t. | - | - | 6/6 |
| **HCN2 I177A** | - 96 ± 0.6 ^n.s.^ | 6.7 ± 0.6 | 18 | n.t. | n.t. | - | - | 18/18 |
| **HCN2 I177D** | n.d | n.d. | 13 | n.t. | n.t. | - | - | 0/13 |
| **HCN2 I177G** | n.d | n.d. | 18 | n.t. | n.t. | - | - | 0/18 |
| **HCN2 I176V** | - 94.9 ± 0.7 ^n.s.^ | 5.1 ± 0.7 | 5 | n.t. | n.t. | - | - | 5/5 |
| **HCN2 I176D** | n.d | n.d | 10 | n.t. | n.t. | - | - | 0/10 |
| **HCN2 I176A** | n.d | n.d | 35 | n.t. | n.t. | - | - | 10/35 |
| **HCN2 L250A** | n.d | n.d | 27 | n.t. | n.t. | - | - | 6/27 |
|  |  |  |  |  |  |  |  |  |
| **HCN2 wt** | - 94.2 ± 0.7 | 5.9 ± 0.7 | 29 | - 78.3 ± 0.6 ^§^ | 6.7 ± 0.5 | 21 | + 15. ± 0.9 | 50/50 |
| **HCN2 F151W** | - 93.7 ± 0.7 ^n.s.^ | 6.2 ± 0.8 | 5 | - 79.5 ± 0.6 ^§^ | 6.8 ± 0.4 | 5 | + 14.2 ± 0.9 ^n.s.^ | 10/10 |
| **HCN2 F151M** | - 92.3 ± 0.5 ^n.s.^ | 6 ± 0.5 | 5 | - 79.2 ± 0.7 ^§^ | 6.8 ± 0.7 | 5 | + 13.1 ± 0.9 ^n.s.^ | 10/11 |
| **HCN2 F151V** | - 90.2 ± 0.7 * | 5.8 ± 0.7 | 9 | - 75.5 ± 0.6 ^§^ | 6.7 ± 0.6 | 5 | + 14.7 ± 0.9 ^n.s.^ | 14/14 |
| **HCN2 F151A** | - 81.6 ± 0.8 * | 6.5 ± 0.4 | 8 | - 64.9 ± 0.5 ^§^ | 7.3 ± 0.8 | 7 | + 16.7± 0.9 ^n.s.^ | 15/21 |
| **HCN2 F151E** | - 59.9 ± 1.1 * | 4.9 ± 0.7 | 3 | - 60.1 ± 1.3 ^n.s.^ | 4.5 ± 0.9 | 3 | -0.2 ± 1.7 * | 6/31 |
| **HCN2 F151R** | -92.2 ± 0.4 ^n.s.^ | 5.6 ± 0.5 | 6 | -76.6 ± 1.2^§^ | 5.7 ± 0.6 | 4 | + 15.6 ± 1.2 ^n.s.^ | 10/24 |
| **HCN2 F151K** | n.d | n.d | 21 | n.d | n.d | - | - | 0/21 |
| **HCN2 F151D** | n.d | n.d | 22 | n.d | n.d | - | - | 0/26 |
|  |  |  |  |  |  |  |  |  |
| **HCN2 wt** | - 96.7 ± 1.2 | 6.3 ± 0.5 | 15 | - 80.2 ± 1.5 ^§^ | 6.8 ± 0.7 | 13 | + 16.7 ± 1.9 | 28/28 |
| **HCN2 K464A** | - 87.5 ± 0.5 * | 5 ± 0.4 | 10 | - 71.8 ± 0.8 ^§^ | 5.6 ± 0.3 | 7 | + 15.7 ± 0.9 ^n.s.^ | 17/17 |
| **HCN2 E478A** | - 115.3 ± 0.7 * | 7.4 ± 0.5 | 7 | - 101.8 ± 0.5 ^§^ | 7.7 ± 0.4 | 6 | + 13.5 ± 0.9 ^n.s.^ | 13/13 |
| **HCN2 K464A-E478A** | - 100.6 ± 0.9 ^n.s.^ | 5.9 ± 0.4 | 8 | - 98.3 ± 1.3 ^n.s.^ | 6.9 ± 0.5 | 8 | + 2.3 ± 1 * | 16/16 |
| **HCN2 K464A-E478A^#^** |  |  |  | -97.4 ± 1.4 ^n.s.^ | 6.2 ± 0.9 | 3 | + 3.2 ± 1.6 * | 3/3 |
|  |  |  |  |  |  |  |  |  |
| **HCN2 wt** | -98.3 ± 1.1 | 5.9 ± 0.5 | 19 | n.t. | n.t. | - | - | 19/19 |
| **HCN2 R154A** | -90.6 ± 0.8 * | 6.3 ± 0.4 | 11 | n.t. | n.t. | - | - | 11/11 |
| **HCN2 E478A** | -115.3 ± 0.7 * | 7.4 ± 0.5 | 7 | n.t. | n.t. | - | - | 7/7 |
| **HCN2 R154A-E478A** | -110 ± 1 * | 6.6 ± 0.6 | 7 | n.t. | n.t. | - | - | 7/7 |

**Supplementary file 1B**

Fitting parameters of the activation curves in HCN1 and HCN4.

From left to right: half-activation voltage (V_1/2_), inverse slope factor (k) obtained by fitting data to a Boltzmann function (Material and Methods) in absence or presence of cAMP; n= number of cell tested in each condition; cAMP-induced shift in V_1/2_; number of cells that expressed a measurable HCN current.

*p<0.05 by One-way ANOVA with Fisher’s test compared to wild-type HCN1 or HCN4; ^§^p<0.05 by Student’s T-test compared to control condition (without cAMP); n.s. not statistically different; n.t. not tested; n.d. not detectable. cAMP concentration used for HCN1 and HCN4 was 15 µM and 30 µM respectively.

|  | **V_1/2_ (mV)**  **± SEM** | **k (mV)**  **± SEM** | **n** | **V_1/2_ (mV)**  **± SEM with cAMP** | **k (mV)**  **± SEM with cAMP** | **n** | **V_1/2_ shift due to cAMP (mV)**  **± SEM** | **Number of cells expressing current** |
| --- | --- | --- | --- | --- | --- | --- | --- | --- |
| **HCN1 wt** | - 72.9 ± 0.5 | 6.9 ± 0.3 | 5 | n.t. | n.t. | - | - | 5/5 |
| **HCN1 I135A** | - 71.7 ± 0.4 ^n.s.^ | 6.2 ± 0.3 | 5 | n.t. | n.t. | - | - | 5/5 |
| **HCN1 I135G** | n.d. | n.d. | 10 | n.t. | n.t. | - | - | 0/10 |
|  |  |  |  |  |  |  |  |  |
| **HCN1 wt** | - 72.4 ± 0.8 | 6.7 ± 0.3 | 5 | n.t. | n.t. | - | - | 5/5 |
| **HCN1 I134V** | - 72.4 ± 0.5 ^n.s.^ | 5.5 ± 0.4 | 6 | n.t. | n.t. | - | - | 6/6 |
| **HCN1 I134A** | n.d. | n.d. | 27 | n.t. | n.t. | - | - | 0/27 |
|  |  |  |  |  |  |  |  |  |
| **HCN1 wt** | - 73 ± 0.4 | 7 ± 0.4 | 5 | n.t. | n.t. | - | - | 5/5 |
| **HCN1 F109A** | - 66 ± 0.3 * | 7.5 ± 0.3 | 6 | n.t. | n.t. | - | - | 6/20 |
| **HCN1 F109E** | n.d. | n.d. | 22 | n.t. | n.t. | - | - | 0/22 |
|  |  |  |  |  |  |  |  |  |
| **HCN1 wt** | - 72.2 ± 0.4 | 7.7 ± 0.3 | 13 | - 71.4 ± 0.9 ^n.s.^ | 7.4 ± 0.5 | 4 | + 0.8 ± 0.5 | 17/17 |
| **HCN1 R549E** | - 80.9 ± 0.3 * | 6.2 ± 0.2 | 5 | n.t. | n.t. | - | - | 5/5 |
| **HCN1 K422A-E436A** | - 78.6 ± 0.4 * | 6.9 ± 0.4 | 11 | - 79.7 ± 0.4 ^n.s.^ | 6.2 ± 0.4 | 4 | + 1.1 ± 0.5 ^n.s.^ | 15/15 |
| **HCN1 K422A-E436A**  **-R549E** | - 79.7 ± 0.4 * | 6.2 ± 0.4 | 10 | n.t. | n.t. | - | - | 10/11 |
|  |  |  |  |  |  |  |  |  |
| **HCN4 wt** | - 102.4 ± 1.9 | 10.8 ± 0.4 | 6 | - 80.5 ± 1.7 ^§^ | 10 ± 1 | 4 | + 21.9 ± 2.5 | 10/10 |
| **HCN4 K543A-E557A** | - 101.1 ± 1.4 ^n.s.^ | 10.3 ± 0.5 | 8 | - 100.3 ± 0.6 ^n.s.^ | 11 ± 0.7 | 7 | + 0.8 ± 1.5 * | 15/15 |

**Supplementary file 1C**

Number of molecules for each simulation of molecular dynamics performed on HCN1.

POPC: 1-palmitoyl-2-oleoyl-glycero-3-phosphocholine; TIP3P: water model; K: K^+^ ion; CL: Cl^-^ ion

| System | Total Atoms | HCN1 | POPC | TIP3P | K | CL |
| --- | --- | --- | --- | --- | --- | --- |
| wt | 297219 | 1 | 487 | 66415 | 180 | 184 |
| F109W | 297883 | 1 | 491 | 66453 | 179 | 183 |
| F109M | 297163 | 1 | 491 | 66453 | 179 | 183 |
| F109V | 207883 | 1 | 487 | 66416 | 180 | 184 |
| F109A | 297834 | 1 | 491 | 664473 | 179 | 183 |
| F109E | 297855 | 1 | 491 | 66448 | 179 | 182 |
