# Supplementary material for: The HCN domain couples voltage gating and cAMP response in hyperpolarization-activated cyclic nucleotide-gated channels
Source: eLife. 2019 Nov 26;8:e49672. doi: 10.7554/eLife.49672 (PMC6894927; doi:10.7554/eLife.49672)
Supplement: Transparent reporting form [file elife-49672-transrepform.docx]

***eLife’s* transparent reporting form**

We encourage authors to provide detailed information *within their submission* to facilitate the interpretation and replication of experiments. Authors can upload supporting documentation to indicate the use of appropriate reporting guidelines for health-related research (see [EQUATOR Network](http://www.equator-network.org/%20)), life science research (see the [BioSharing Information Resource](https://biosharing.org/)), or the [ARRIVE guidelines](http://www.plosbiology.org/article/info:doi/10.1371/journal.pbio.1000412) for reporting work involving animal research. Where applicable, authors should refer to any relevant reporting standards documents in this form.

If you have any questions, please consult our Journal Policies and/or contact us: [editorial@elifesciences.org](mailto:editorial@elifesciences.org).

**Sample-size estimation**

- You should state whether an appropriate sample size was computed when the study was being designed
- You should state the statistical method of sample size computation and any required assumptions
- If no explicit power analysis was used, you should describe how you decided what sample (replicate) size (number) to use

Please outline where this information can be found within the submission (e.g., sections or figure legends), or explain why this information doesn’t apply to your submission:

No explicit power analyses were used to calculate sample sizes. The number of replicates was guided by the variability between individual measurements and the accepted number of repeats in the field.

Electrophysiology: for patch-clamp experiments in whole cell configuration we used as a sample size between 10-15 cells, never less than 5, from at least two independent transfections, usually 3 or more. The exact number of experiments for each construct that was tested is reported in Table S1 and S2.

MD simulation and LRT: does not apply/See replicates

**Replicates**

- You should report how often each experiment was performed
- You should include a definition of biological versus technical replication
- The data obtained should be provided and sufficient information should be provided to indicate the number of independent biological and/or technical replicates
- If you encountered any outliers, you should describe how these were handled
- Criteria for exclusion/inclusion of data should be clearly stated
- High-throughput sequence data should be uploaded before submission, with a private link for reviewers provided (these are available from both GEO and ArrayExpress)

Please outline where this information can be found within the submission (e.g., sections or figure legends), or explain why this information doesn’t apply to your submission:

Elettrophysiology: we usually repeat the same experiment with different batches of cells (>2) and with different plasmid DNA preparations >2).

The number of replicates for each experiment is stated in Table S1 and S2.

LRT computation was done once per position, as the robustness of this model has been justified in a previous study (Gross et aĺ. 2017).

MD: no replicas were performed. MD simulations are ergodic. Statistical properties can be deduced from a single, long trajectory because the time average of one simulation equals the ensemble average.

**Statistical reporting**

- Statistical analysis methods should be described and justified
- Raw data should be presented in figures whenever informative to do so (typically when N per group is less than 10)
- For each experiment, you should identify the statistical tests used, exact values of N, definitions of center, methods of multiple test correction, and dispersion and precision measures (e.g., mean, median, SD, SEM, confidence intervals; and, for the major substantive results, a measure of effect size (e.g., Pearson's r, Cohen's d)
- Report exact p-values wherever possible alongside the summary statistics and 95% confidence intervals. These should be reported for all key questions and not only when the p-value is less than 0.05.

Please outline where this information can be found within the submission (e.g., sections or figure legends), or explain why this information doesn’t apply to your submission:

Electrophysiology: The statistical tests used are stated, together with the results, in Table S1 and S2. Activation curves were evaluated by comparing the mean V_1/2_ values derived by the Boltzmann fitting using one-way ANOVA followed by Fisher’s test. Significance level was set to p=0.05. Student’s unpaired *t* test was used to compare the treated (with cAMP) and untreated (without cAMP) conditions. Significance level was set to p=0.05

MD simulation: Fig 3C has confidence intervals and outliers and outliers in the boxplot

(For large datasets, or papers with a very large number of statistical tests, you may upload a single table file with tests, Ns, etc., with reference to sections in the manuscript.)

**Group allocation**

- Indicate how samples were allocated into experimental groups (in the case of clinical studies, please specify allocation to treatment method); if randomization was used, please also state if restricted randomization was applied
- Indicate if masking was used during group allocation, data collection and/or data analysis

Please outline where this information can be found within the submission (e.g., sections or figure legends), or explain why this information doesn’t apply to your submission:

Does not apply to our study

**Additional data files (“source data”)**

- We encourage you to upload relevant additional data files, such as numerical data that are represented as a graph in a figure, or as a summary table
- Where provided, these should be in the most useful format, and they can be uploaded as “Source data” files linked to a main figure or table
- Include model definition files including the full list of parameters used
- Include code used for data analysis (e.g., R, MatLab)
- Avoid stating that data files are “available upon request”

Please indicate the figures or tables for which source data files have been provided:

LRT computations (Figs. 5 and S5): R package, R code, input PDB structure

MD simulation (Fig 3C and 3D): row data and files to reproduce the MD simulation
